# Notes and News

**Published:** 1903-05-09

**Authors:** 


### Notes and News.

Mr. Henry James Tressider, who has retired from the Secretaryship of the National Orthopædic Hospital, was formerly a clerk in the office of the Surgical Aid Society, not the Secretary as Mr. Tressider in mistake informed us.

We are informed that it is not at present the intention of the Senate of the University of London to extend the same privileges to the medical graduates as have been granted to graduates in the other faculties of certain foreign and colonial universities with regard to exemption from the lower examinations previously to taking the higher London degrees.

At the meeting to be held on Thursday (14th) of the British Gynæcological Society at 20 Hanover Square, W., at 8 p.m., specimens will be shown by Dr. Macnaughton Jones and others. There will be an adjourned discussion on Mr. Bowreman Jessett's paper, "Some Rare Complications accompanying Ectopic Gestation;" and on that of Dr. C. H. R. Routh on "Some Directions and Avenues through which probably a more successful Treatment of Cancer may result and perhaps cure."

A meeting of the Childhood Society will be held at 7 St. James's Square, S.W., on Monday, May 11th, at 3.30 p.m., when an address will be delivered by Professor John Edgar, M.A., etc., Professor of Education, St. Andrew's University, on "The Universities and the Scientific Study of Children, with special reference to the Teaching Profession." Admission is by ticket only, to be obtained of the Hon. Secretary of the society, Parkes Museum, Margaret Street, W.

Considerable interest attaches to the approaching garden party at Marlborough House to which their Royal Highnesses the Prince and Princess of Wales, as Grand President and Lady Grand President, are inviting the presidents, lady presidents, vice-presidents, and other workers for the League of Mercy. The garden party will take place at 4 p.m. on Friday, May 22nd, and it is understood that the presidents and lady presidents of the League will be received by the Prince and Princess of Wales in the afternoon. The League has already been instrumental in contributing £22,000 to King Edward's Hospital Fund, and a "Samaritan" branch of its work is in process of organisation. The offices of the League are at 29 Southampton Street, Strand, W.C.

The Scientific Press will shortly issue a small handbook entitled, "Practical Guide to Surgical Bandaging and Dressing," by Wm. Johnson Smith, F.R.C.S. This work should prove of much value to junior students and nurses as it contains very full explanations of the most modern scientific methods in surgical work.

In connection with the Printers' Pension Corporation, whose anniversary festival is held in June, a special matinee performance will take place at the Garrick Theatre, Charing Cross Road, W.C., kindly lent by Mr. Arthur Bourchier, on Tuesday, June 9th, the proceeds of which will go to the funds of the excellent charity, which is noted for its economical management and beneficent work in aiding the less fortunate in the printing trades. The Princess Louise has consented to become a patroness, and she is supported by a number of influential ladies. On behalf of the same institution the "Printers' Pie," an interesting book to form a festival souvenir of the Corporation, is being prepared by Mr. W. Hugh Spottiswoode, who presides at the annual festival dinner. Practically all the work connected with the production of the souvenir is being gratuitously given, and amongst the contributors to the work are Miss Braddon, Miss Marie Corelli, Ouida, Hall Caine, B. L. Farjeon, Frederic Harrison, T. P. O'Connor, Israel Zangwill, and others. The "Printers' Pie," will be sold to the public at 2s. 6d. net.

The Lord Mayor's committee of inquiry into the affairs of St. Bartholomew's Hospital held a meeting on May 5th at the Mansion House, the Lord Mayor presiding. The meeting was held for the purpose of receiving the report of the sub-committee on administration and finance, of which Sir Thomas Jackson was the chairman. After a lengthened consideration of the report the following resolution was unanimously agreed to:—"That this committee, having carefully considered the report of the sub-committee on the financial and administrative management of the hospital (which is summarised in the "conclusions" given below) are of opinion that the governors have completely vindicated the reputation, character, and administration of the hospital, and are fully justified in appealing to the public for funds to enable them to utilise the land acquired from Christ's Hospital, and to provide the new buildings urgently necessary to bring the hospital up to modern requirements in all respects." The conclusions at which the sub-committee had unanimously arrived were summarised in the report as follows:—"(1) That the hospital is properly and economically administered and that an increase rather than a reduction of expenditure must be looked for: (2) that any prospective increase of rental will be more than absorbed by the deficit caused by the purchase of land from Christ's Hospital, and consequently (3) that no part of the outlay that will have to be incurred for new buildings can be provided out of the hospital's funds except by additional borrowing that would entail a further loss of income." In March the Lord Mayor's committee passed a resolution to the effect that it was desirable in the public interest to retain St. Bartholomew's Hospital on its present site. It yet remains for the building sub-committee to consider the plans for the new buildings.

